# Swelling Behavior, Biocompatibility, and Controlled Delivery of Sodium–Diclofenac in New Temperature-Responsive P(OEGMA/OPGMA) Copolymeric Hydrogels

**DOI:** 10.3390/gels11030201

**Published:** 2025-03-14

**Authors:** Zorana Rogic Miladinovic, Maja Krstic, Edin Suljovrujic

**Affiliations:** Vinca Institute of Nuclear Sciences, National Institute of the Republic of Serbia, University of Belgrade, Mike Petrovica Alasa 12-14, P.O. Box 522, 11001 Belgrade, Serbia; zoranar@vin.bg.ac.rs (Z.R.M.); majamicic@vin.bg.ac.rs (M.K.)

**Keywords:** hydrogel, gamma radiation, controlled drug release, propylene glycol methacrylate, ethylene glycol methacrylate, *VPTT*

## Abstract

This study investigates the synthesis and properties of innovative poly(oligo(alkylene glycol)) methacrylate hydrogels synthesized via gamma radiation-induced copolymerization and the crosslinking of oligo(ethylene glycol) methacrylate (OEGMA) and oligo(propylene glycol) methacrylate (OPGMA) at varying mole fractions. Our primary objective is to investigate the impact of copolymerization on the swelling properties of P(OEGMA/OPGMA) hydrogels compared to their homopolymeric counterparts, namely, POEGMA and POPGMA, which exhibit distinct volume phase transition temperatures (*VPTT*s) of around 70 and 13 °C, respectively, under physiological conditions. To this end, a comprehensive library of smart methacrylate-based hydrogel biomaterials was developed, featuring detailed data on their swelling behavior across different copolymer molar ratios and physiological temperature ranges. To achieve these objectives, we conducted swelling behavior analysis across a wide range of temperatures, assessed the pH sensitivity of hydrogels, utilized scanning electron microscopy for morphological characterization, performed in vitro biocompatibility assessment through cell viability and hemolysis assays, and employed diclofenac sodium as a model drug to control drug delivery testing. Our findings demonstrate that the newly synthesized P(OEGMA_40_/OPGMA_60_) copolymeric hydrogel exhibits desirable characteristics, with *VPTT* close to the physiological temperatures required for controlled drug delivery applications.

## 1. Introduction

Hydrogels have garnered significant attention for their exceptional capabilities as matrices for drug delivery systems. Their water-retentive and porous structures, which closely mimic human tissue, facilitate the effective encapsulation and controlled release of therapeutic agents [1]. This intrinsic property allows for the precise modulation of drug release kinetics, making hydrogels adaptable for various delivery routes, including oral, transdermal, rectal, and buccal [2]. Advanced hydrogels, particularly those responsive to temperature and pH changes, offer enhanced control over drug release by reacting to physiological conditions, thus improving treatment efficacy and minimizing side effects [3,4,5,6,7,8].

One notable challenge in in vitro drug delivery from hydrogels is the burst effect, where a significant portion of the encapsulated drug is released rapidly due to its proximity to the hydrogel surface or loosely bound regions. This initial burst can compromise the effectiveness of drug delivery systems by leading to uncontrolled release and reduced therapeutic efficacy [9]. Strategies to achieve a more sustained and controlled release profile include optimizing polymer composition, adjusting crosslinking density, incorporating release modifiers, employing multi-layered hydrogel systems, and potentially increasing the pressure during compression to enhance material density, which can further extend the release profile [10,11,12,13].

In the realm of smart hydrogels, methacrylate-based hydrogels have emerged as a significant category, with copolymerized gelatin hydrogels gaining prominence. Gelatin, derived from collagen, is valued for its thermo-reversible gel formation, transparency, and melting point at body temperature [14,15]. While gelatin-based hydrogels show versatility in applications such as wound management and tissue engineering [16], they often face challenges related to thermal and mechanical instability, variable degradation rates, and potential immune responses.

Synthetic polymer hydrogels, on the other hand, offer an alternative by providing precise control over mechanical properties and other characteristics, although they typically lack inherent bioactivity [17]. This underscores the potential advantages of synthetic hydrogels in overcoming some limitations of natural monomer-based systems.

Temperature-sensitive monomers, such as those containing poly(ethylene glycol) (PEG), are widely explored due to their favorable thermodynamic properties and biocompatibility [18,19]. Monomers like PEG methacrylate (PEGMA), with a hydrophobic polymethacrylate backbone and hydrophilic PEG side chains, are utilized in developing smart materials for biomedical applications [20,21]. Despite PEGMA’s recognized biocompatibility and water retention capabilities, it has been noted for attracting contaminants, which diminishes its bio-repulsive properties [22].

In contrast, poly(propylene glycol) (PPG) methacrylate (PPGMA) offers distinct advantages. The hydrophobic CH_3_ group in PPG contributes to its hydrophobicity and lowers the volume transition temperature (*VPTT*), resulting in reduced swelling and minimal contamination adhesion. These properties suggest that PPGMA could be used to create amphiphilic coatings with improved contaminant repellency [22]. Despite this promising attribute, PPGMA and its analogs remain underexplored, leaving their potential applications largely unknown. Previous research [23] demonstrated high sol-gel conversion rates and significant temperature sensitivity in homopolymeric PPGMA. However, its swelling behavior and temperature sensitivity have been observed at temperatures well below room temperature, which restricts its potential for biomedical applications.

The primary objective of this study was to develop a comprehensive library of smart methacrylate-based hydrogel biomaterials, with detailed data on their swelling behavior across various copolymer molar ratios in the range of physiological temperatures. To achieve this, we employed gamma radiation to copolymerize and crosslink methacrylate monomers functionalized with ethylene glycol (EG) and propylene glycol (PG) units in varying molar ratios.

This method was chosen due to the possibility of performing their sterilization simultaneously with the synthesis of copolymer hydrogels, which avoids the addition of initiators and activators, thus obtaining hydrogels free from impurities, making them suitable for biomedical use.

The synthesized hydrogels were characterized using physicochemical methods, i.e., gravimetric and morphological analyses, to explore the relationship between their swelling properties and morphology. In vitro biological assays assessed the hydrogels’ biocompatibility, and an in vitro drug release study was conducted to evaluate the hydrogels as potential carriers for controlled drug delivery, using diclofenac sodium as the model drug.

## 2. Results and Discussion

### 2.1. Swelling Analysis

**Figure 1:**
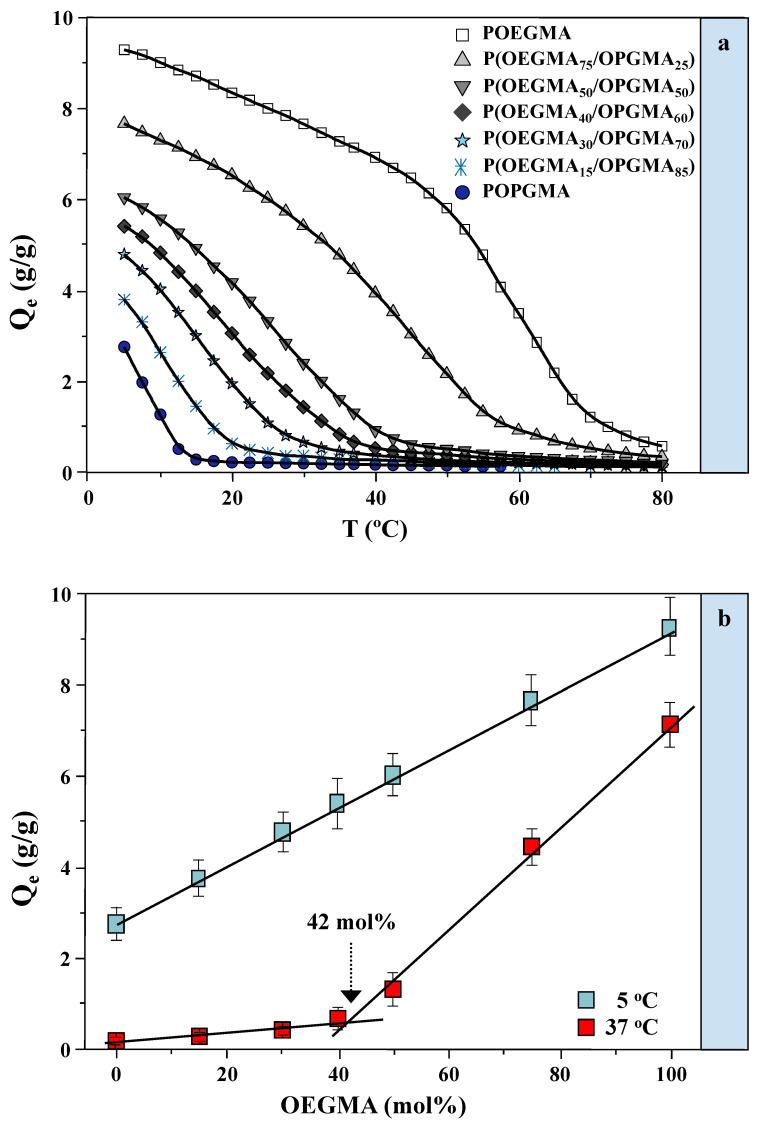
(**a**) Equilibrium swelling profiles for POEGMA and POPGMA homopolymeric hydrogels and P(OEGMA_75_/OPGMA_25_), P(OEGMA_50_/OPGMA_50_), P(OEGMA_40_/OPGMA_60_), P(OEGMA_30_/OPGMA_70_), and P(OEGMA_15_/OPGMA_85_) copolymeric hydrogels in pH 7.4 buffer solution as a function of temperature; (**b**) effect of OEGMA monomer content in P(OEGMA/OPGMA) copolymeric hydrogels on equilibrium swelling degree at two temperatures: 5 °C (for drug uptake) and 37 °C (for drug release).

A swelling study of the synthesized hydrogels performed in a pH 7.4 buffer solution and a wide range of temperatures is presented in Figure 1a. Equilibrium swelling degrees of the hydrogels varied with the monomer composition.

The pure POPGMA hydrogel exhibited poor swelling at the initial measurement temperature, while adding the OEGMA content enhanced the swelling capacity. The results revealed that at 5 °C, the homopolymeric POPGMA hydrogel had an equilibrium swelling degree (*Q_e_*) of about 2.9, whereas the homopolymeric POEGMA hydrogel had a *Q_e_* of approximately 9.3. Copolymeric hydrogels with different OEGMA-to-OPGMA ratios displayed *Q_e_* values between these extreme values. As the proportion of the more hydrophilic OEGMA component in the P(OEGMA/OPGMA) copolymer increases, the *Q_e_* also rises, reflecting the greater hydrophilicity of OEGMA compared to OPGMA. All hydrogels showed negative temperature sensitivity, with *Q_e_* decreasing as the temperature increased. At 40 °C, a temperature relevant for biological applications, significant differences in the *Q_e_* were observed, particularly in hydrogels with higher OEGMA content. These differences diminished with increasing temperature, yet they remained notably distinct in hydrogels with higher OEGMA concentrations. Only at temperatures exceeding 75 °C did the *Q_e_* for all hydrogels fall below 0.5, a threshold beyond which the temperature becomes less relevant for biomedical applications.

To analyze the influence of the share of monomers on the degree of equilibrium swelling, in the physiological pH, and at the temperatures of drug absorption (5 °C) and drug release (37 °C), diverse trends were observed (Figure 1b). The findings at 5 °C demonstrate a clear linear relationship between the equilibrium swelling degree and the proportion of OEGMA monomer present within the hydrogel. In contrast, the correlation at 37 °C reveals a more intricate pattern. Specifically, the equilibrium swelling degree shows a slight increase for hydrogels with lower concentrations of OEGMA monomer. However, once the proportion of OEGMA exceeds 42 mol%, a notable sharp increase in the equilibrium swelling degree is observed, signifying an enhanced hydrogel capacity and improved drug uptake capabilities. Notably, a significant difference in the equilibrium swelling degree was observed for hydrogels with 42 mol% OEGMA when measured at two temperatures relevant to drug delivery and uptake. At 37 °C, which simulates drug delivery conditions, *Q_e_* is around 0.7, whereas at 5 °C, representing drug uptake conditions, *Q_e_* is approximately 5.2. Within the presented results, this disparity is largest for the copolymeric hydrogel, at about 42 mol% OEGMA, suggesting that this hydrogel possesses an increased ability to uptake and deliver larger drug quantities.

**Figure 2:**
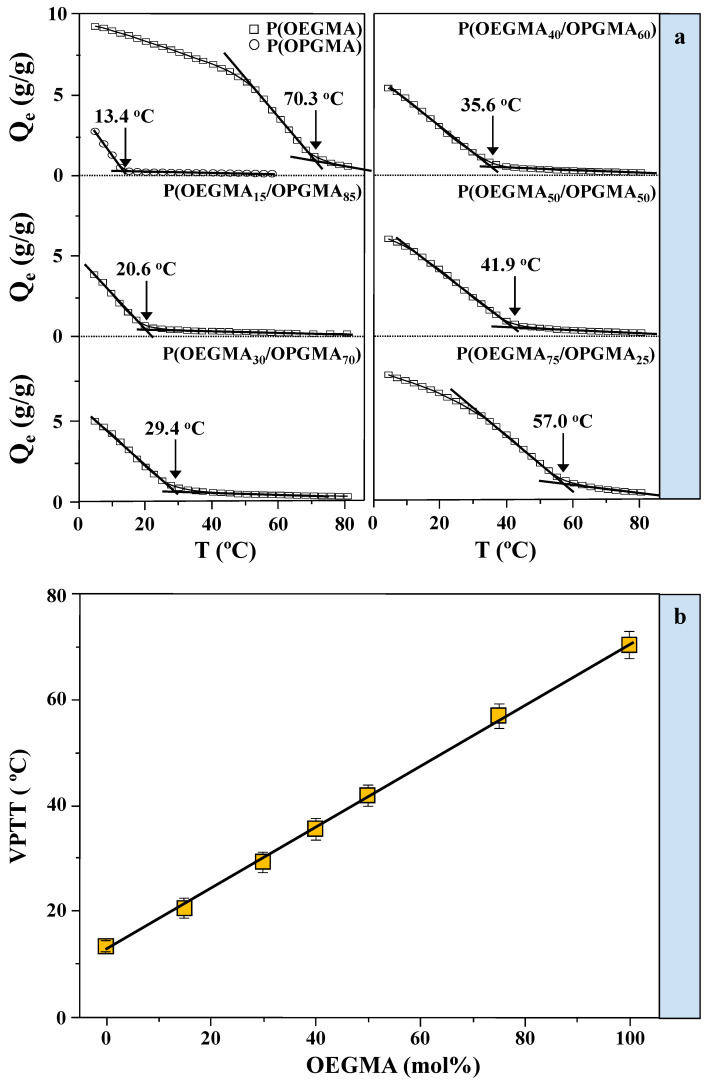
(**a**) Equilibrium swelling profiles of POEGMA and POPGMA homopolymeric hydrogels, along with P(OEGMA_15_/OPGMA_85_), P(OEGMA_30_/OPGMA_70_), P(OEGMA_40_/OPGMA_60_), P(OEGMA_50_/OPGMA_50_), and P(OEGMA_75_/OPGMA_25_), copolymeric hydrogels, in pH 7.4 buffer solution as a function of temperature, with volume phase transition values indicated; (**b**) variation in *VPTT* of the investigated copolymeric hydrogels with OEGMA content (mol%) in pH 7.4 buffer solution.

On the other hand, by considering the equilibrium swelling degrees of hydrogels versus temperature (Figure 2a), it can be noticed that apart from showing a significantly higher degree of swelling, hydrogels with a 100 and 75 mol% share of OEGMA monomer also show a similar trend, with weak temperature dependence up to about 40 and 30 °C, respectively, as well as a milder decrease in the equilibrium degree of swelling with a further increase in temperature. As a consequence, the temperatures of volume phase transitions are at values (70.3 and 57 °C) far above the physiological temperatures, i.e., temperatures of importance for biomedical applications. Likewise, hydrogels with a higher share of OPGMA have lower degrees of swelling, *VPTT* values lower than physiological temperatures, and a sharper temperature transition. Further, a gradual increase in the share of OEGMA led not only to an increase in the degree of swelling but also to a shift of the phase transition to higher temperatures. It was observed that the *VPTT* values for this set of hydrogels are 13.7 and 70.3 °C for homopolymeric hydrogels while, depending on the share of monomers in the copolymeric hydrogel, *VPTT* values are found in the temperature range from 20.6 to 57.0 °C. The two copolymeric hydrogels, P(OEGMA_40_/OPGMA_60_) and P(OEGMA_50_/OPGMA_50_), exhibit *VPTT* values within the temperature range of mild hypothermia (35.6 °C) [24] and moderate hyperthermia (41.9 °C) [25], respectively, making them promising materials for biomedical applications.

Moreover, by determining the *VPTT* for the examined hydrogels, we gained detailed insights into the temperature behavior of the system as it relates to the molar fraction of OEGMA (or OPGMA) in the P(OEGMA/OPGMA) copolymeric hydrogels (Figure 2b). The observed linear relationship suggests that the required share of comonomers for hydrogel synthesis can be easily determined based on the temperature at which the volume change occurs, facilitating the selection of the monomer ratio for targeted applications. This illustrates a clear correlation between the structure and properties, wherein P(OEGMA/OPGMA) hydrogels exhibit a consistent rise in *VPTT* as the molar percentage of OEGMA within the copolymer increases. A comparable linear relationship between the *VPTT* and the proportion of the incorporated component was already observed in certain copolymeric hydrogels [26,27]. Still, this linear relationship also suggests an optimal composition of copolymeric hydrogels containing approximately 42 mol% of OEGMA monomer for biomedical applications, given that their *VPTT* falls within physiological limits, around 37 °C.

### 2.2. Morphological Analysis

**Figure 3:**
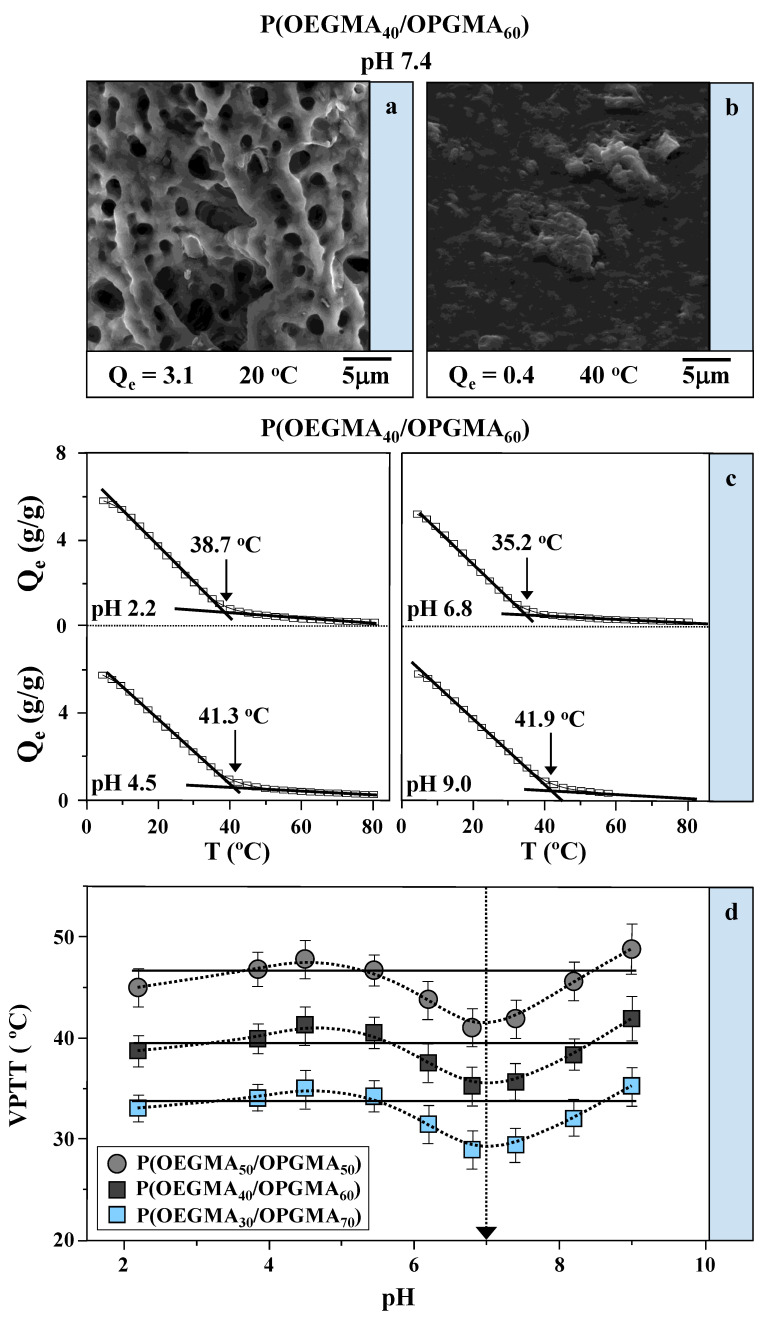
Cross-section microstructures of P(OEGMA_40_/OPGMA_60_) copolymeric hydrogel swollen to equilibrium at (**a**) 20 °C (below *VPTT*) and (**b**) 40 °C (above *VPTT*) in pH 7.4 buffer solution before lyophilization; (**c**) equilibrium swelling profiles for P(OEGMA_40_/OPGMA_60_) copolymeric hydrogels in pH 2.2, 4.5, 6.8, and 9.0 buffer solution as a function of temperature, with volume phase transition values indicated; (**d**) *VPTT* of P(OEGMA_50_/OPGMA_50_), P(OEGMA_40_/OPGMA_60_), and P(OEGMA_30_/OPGMA_70_) copolymeric hydrogels as a function of buffer solution pH. The horizontal lines are presented to ensure visual clarity of hydrogels’ behaviour around the pH 7 (emphasised with the dotted arrow).

The morphological analysis was conducted using scanning electron microscopy (SEM) on P(OEGMA_40_/OPGMA_60_) hydrogel specimens that were pre-swelled in pH 7.4 buffer solution to temperatures of 20 and 40 °C, temperatures far below and above the *VPTT*, which is 35.6 °C. Afterward, the samples were freeze-dried. SEM micrographs depicted a reduction in porosity correlating with an increase in temperature, as shown in Figure 3a,b. For the sample recorded at 20 °C, a pronounced spongy porous structure was observed, with a relatively uniform distribution of pores, which is characteristic of hydrogels with a higher degree of swelling (*Q_e_* = 3.1). Conversely, at 40 °C, the copolymeric hydrogel exhibited a compact, non-porous structure. This indicated a lower equilibrium swelling degree, which decreased to no more than 0.4. These observed microstructural changes emphasize the influence of temperature not only on the swelling capacity and volume phase transition but also on the porosity control of hydrogels.

### 2.3. Assessment of pH Sensitivity

The swelling of the hydrogels was tested in various media with pH values ranging from highly acidic to basic for two months. It was found that during this time, the hydrogels maintained their shape and structural integrity, showing no signs of degradation. Based on Figure 3c, no significant difference is observed in the equilibrium degree and dynamics of swelling of P(OEGMA_40_/OPGMA_60_) copolymeric hydrogels measured in four different buffer solutions. However, there is a considerable distinction between the temperatures at which the volume phase transition appears, 38.7, 41.3, 35.2, and 41.9 °C for pH values 2.2, 4.5, 6.8, and 9.0, respectively. For pH 7.4, the *VPTT* is 35.6 °C, according to Figure 2a. The results indicate that the *VPTT* values at pH 6.8 are markedly lower than those at lower and higher pH values, demonstrating distinct differences in behavior outside this pH range. Although the studied hydrogels do not contain pH-sensitive groups, namely, they belong to the group of non-ionic hydrogels, the resulting changes in sensitivity are most likely a consequence of the indirect contact of ions and alkylene glycol units, influencing the redistribution of hydrogen bonds within the system and consequently influencing the weakening of the interactions between polymer and water [28,29]. It was somewhat anticipated that methacrylate hydrogels would act as described [30,31]. Likewise, in Figure 3d, it is evident that *VPTT* values, plotted against pH, show consistent trends across the three copolymeric compositions studied. *VPTT* values are higher in acidic and basic buffer solutions compared to neutral pH, highlighting the pH sensitivity of non-ionic methacrylate polymer networks.

### 2.4. Cell Viability

**Figure 4:**
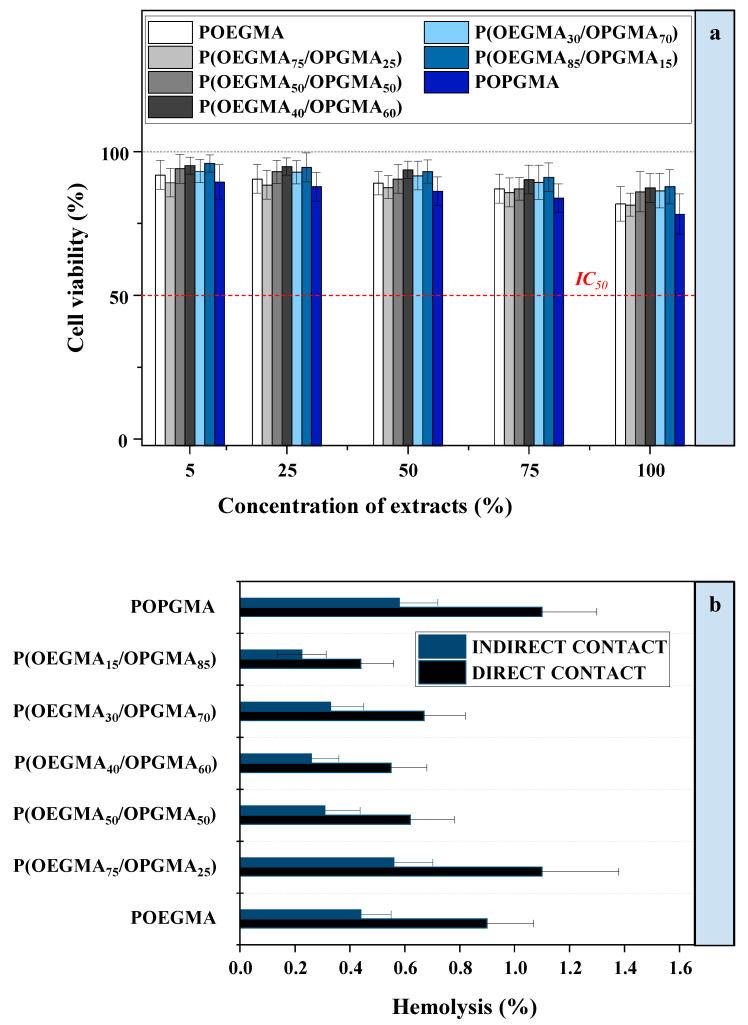
(**a**) Cell viability of POEGMA, P(OEGMA_75_/OPGMA_25_), P(OEGMA_50_/OPGMA_50_), P(OEGMA_40_/OPGMA_60_), P(OEGMA_30_/OPGMA_70_), P(OEGMA_15_/OPGMA_85_), and POPGMA hydrogels as a function of concentration of extracts; (**b**) hemolytic activity of POEGMA, P(OEGMA_75_/OPGMA_25_), P(OEGMA_50_/OPGMA_50_), P(OEGMA_40_/OPGMA_60_), P(OEGMA_30_/OPGMA_70_), P(OEGMA_15_/OPGMA_85_), and POPGMA hydrogels.

To evaluate the cytotoxicity of fabricated hydrogel materials, the in vitro study of biocompatibility using a cytotoxicity assay was performed. The cells isolated from mouse fibroblast (L929) were incubated with different concentrations of hydrogels for 2 h. The relative percentages of the numbers of visible colonies that emerged with various extract concentrations (cell viability) as a result of the interaction of the cells and the hydrogel were estimated and presented in Figure 4a. Although the cell viability value varied independently of the hydrogel composition, all fabricated hydrogels co-cultured with L929 cells showed high viabilities (above 80%). Therefore, it is plausible that the developed hydrogels have excellent cytocompatibility since all cell viability values far exceed 50%, which is considered to be the concentration of the extract required to induce 50% cell mortality [32,33] and the required condition for the hydrogels to possess acceptable cytotoxicity [34,35].

### 2.5. Hemolysis

The evaluation of hemolytic properties is essential for hydrogels that may be employed for biomedical applications, because when exposed to blood, they may cause hemolysis of the blood cells [36]. Hydrogels are defined as hemolytic when they have more than 5% hemolysis, slightly hemolytic for hemolytic values in the range from 2 to 5%, and non-hemolytic when the hemolysis is below 2% [37]. According to the in vitro blood compatibility results (Figure 4b), the differences in hemolysis values are negligible and no dependence on the hydrogel composition is observed. All fabricated hydrogels are hemocompatible, with hemolysis rates under 1.5% for direct contact and below 0.6% for indirect contact, which is below the acceptable limits for hemolysis in both cases. These findings support the notion that P(OEGMA/OPGMA) copolymeric hydrogels are non-hemolytic and suitable for biomedical applications.

### 2.6. Drug Delivery

For every drug, delivery aims to optimize therapeutic use by efficiently transporting and releasing the drug to the intended site within the body while simultaneously minimizing accumulation away from the targeted [38]. Smart hydrogels are of particular interest in controlled-release drug delivery systems due to their unique capacity for tunable time-dependent swelling. The release kinetics of drugs are dependent upon various factors, including the properties of the hydrogel and the drug itself, their interactions, and the specific conditions of release.

**Figure 5:**
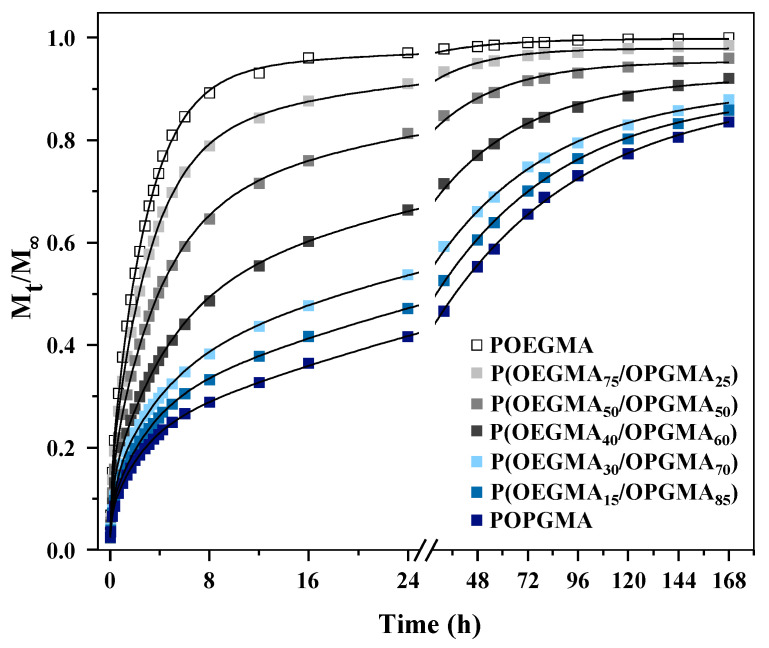
Drug release profiles of diclofenac sodium from POEGMA, P(OEGMA_75_/OPGMA_25_), P(OEGMA_50_/OPGMA_50_), P(OEGMA_40_/OPGMA_60_), P(OEGMA_30_/OPGMA_70_), P(OEGMA_15_/OPGMA_85_), and POPGMA hydrogel matrices into pH 7.40 physiological buffer solutions at 37 °C.

In this study, we present the release profiles of diclofenac sodium (DS) from the P(OEGMA/OPGMA) copolymeric hydrogel matrix, as well as from POEGMA and POPGMA homopolymeric hydrogel matrices, into pH 7.40 physiological buffer solutions at 37 °C, as shown in Figure 5.

The drug was tested over 7 days to better understand the drug release profiles and the performance of the proposed matrix for short and long therapeutic durations. The POEGMA homopolymeric hydrogel displayed a distinct burst effect, releasing DS rapidly within the initial 8 h, followed by a gradual release until the 16th hour, when it reached equilibrium values, after which only minor changes were observed. This behavior is attributed to high *VPTT*, a significant degree of swelling, and porosity values characteristic for hydrogels with more than a 50 mol% share of the OEGMA monomer. In contrast, the POPGMA homopolymeric hydrogel demonstrated sustained release kinetics, devoid of any initial burst effect, suggesting a controlled drug release mechanism.

The copolymerization of OEGMA with the hydrophobic OPGMA monomer effectively altered the burst release characteristics and notably influenced the release profiles of the copolymeric P(OEGMA/OPGMA). By increasing the proportion of OPGMA monomer in the copolymeric hydrogel, convergence toward the profile of the homopolymeric POPGMA hydrogel is observed, indicating that the copolymeric hydrogels successfully underwent the adjustment of vastly different characteristics of drug release carriers: POEGMA and POPGMA. Furthermore, an increase in the proportion of the OEGMA monomer in the copolymeric hydrogel resulted in a higher drug release rate.

The kinetic constant (*k*), diffusion exponent (*n*), and diffusion coefficients for both the early (*D_e_*) and late (*D_l_*) stages of drug release, along with the drug release half-time (*t*_1/2_) indicating the duration to release half of the loaded drug in the xerogel, were derived using Equations (4)–(7), which are outlined in the Experimental section and are presented in Table 1.

The range of *n* values spans from 0.50 to 0.39. All hydrogels exhibit Fickian drug release behavior, whereas a decrease in *n* correlates with a reduction in the proportion of the OEGMA monomer within the copolymeric hydrogel. This implies slower mobility of the DS drug in hydrogels with a higher hydrophobic OPGMA content. Such observations are indicative of a Fickian release mechanism, characteristic of diffusion-controlled delivery systems with cylindrical slab geometry. Moreover, this suggests the potential for altering diffusion properties by adjusting the composition of the copolymeric hydrogel [39].

The diffusion coefficients for early (*D_e_*) and late (*D_l_*) stages of drug release are influenced by the composition of the hydrogel, specifically the ratio of OEGMA to OPGMA monomers. The highest *D_e_* and *D_l_* values are observed for the POEGMA homopolymeric hydrogel and P(OEGMA_75_/OPGMA_25_) copolymeric hydrogel due to their higher content of hydrophilic OEGMA monomer and faster release rates. Conversely, the diffusion coefficients decrease with a decreasing share of the OEGMA monomer, reaching their lowest values for the P(OEGMA_15_/OPGMA_85_) copolymeric hydrogel and POPGMA homopolymeric hydrogel. The swelling and deswelling behavior of the hydrogels correlates with the release process, with higher swelling degrees enhancing and accelerating drug release, and reduced swelling slowing it down. In all samples, the early-time diffusion coefficient *D_e_* exceeds the late diffusion coefficient *D_l_*, which is consistent with the faster release observed in the early phase, attributed to intense pendant chain motions through the polymeric network. Conversely, in the late phase, slower drug diffusion is achieved and, eventually, constant release.

The *D_e_*/*D_l_* ratio, indicative of the balance of drug delivery, decreases with decreasing OEGMA content, from 9.1 for POEGMA homopolymeric and 8.5 for P(OEGMA_75_/OPGMA_25_) copolymeric hydrogels to 2.3 and 2.1 for P(OEGMA_15_/OPGMA_85_) copolymeric and POPGMA homopolymeric hydrogels, respectively. These findings suggest that hydrogel composition plays a crucial role in regulating drug release kinetics, with implications for controlled drug delivery applications.

Significant variations in drug release half-time (*t*_1/2_) are noteworthy, demonstrating the influence of the composition and OPGMA units on the mobility of the DS drug. Our findings reveal that *t*_1/2_ extends from 1.7 h for the POEGMA homopolymeric hydrogel and 2.3 h for the P(OEGMA_75_/OPGMA_25_) copolymeric hydrogel to 29.1 h for the P(OEGMA_15_/OPGMA_85_) copolymeric hydrogel and 38.8 h for the POPGMA homopolymeric hydrogel. This trend implies that hydrogels with a higher proportion of hydrophobic OPGMA monomers in P(OEGMA/OPGMA) copolymeric hydrogels are more suitable for long-term drug delivery applications.

Overall, increasing the proportion of the OEGMA monomer in the hydrogel led to enhanced swelling and higher diffusion coefficients, indicating improved mobility of diclofenac sodium within the hydrogel matrix. In contrast, hydrogels with a higher percentage of the OPGMA monomer exhibited better control over drug release, as demonstrated by the model drug, while also minimizing the burst effect associated with drug delivery. However, these hydrogels showed comparatively lower drug uptake than those enriched with OEGMA. This suggests that while hydrogels with a higher OPGMA content are more effective in sustaining drug release and reducing the burst effect, they are less efficient in promoting initial drug uptake compared to those with a higher OEGMA content. Based on these findings, it is crucial to determine the optimal balance of OEGMA and OPGMA monomers to achieve both efficient drug uptake and controlled, sustained release, tailored to the specific needs of various therapeutic applications. Among the hydrogels investigated, the P(OEGMA_40_/OPGMA_60_) drug carrier demonstrated a significantly slower drug release profile with minimal burst effect, further supporting its potential as an optimal choice for controlled drug delivery.

Considering the afore-mentioned factors and the potential long-term degradability of tested hydrogels, the transdermal and dental route may offer a safer, non-invasive method of administration while maintaining consistent and reliable therapeutic concentrations [40,41].

## 3. Conclusions

In this study, the gamma radiation-synthesized P(OEGMA/OPGMA) copolymeric hydrogels exhibit *VPTT*s positioned between those of the POEGMA and POPGMA homopolymeric hydrogels, providing clear evidence of successful property modulation through copolymerization. Furthermore, plotting *VPTT* against the proportion of OEGMA comonomer reveals a discernible pattern, offering valuable insights into copolymerization outcomes and facilitating predictive modeling for specific applications of hydrogels. The hydrogel with 42 mol% of OEGMA monomer demonstrated the highest potential for drug release under physiological conditions, leading us to select the most analogous hydrogel, P(OEGMA_40_/OPGMA_60_), to investigate its morphology and evaluate its pH sensitivity. The analysis confirms its temperature sensitivity and surprisingly reveals its pH responsiveness. Evaluation of in vitro biocompatibility, encompassing cytocompatibility and hemolytic activity, exposes encouragingly satisfactory biocompatibility for all copolymeric hydrogels, finally underscoring their potential for biomedical applications. It is also noteworthy that all the synthesized hydrogels demonstrated strength; ease of handling; and resistance to brittleness, cracking, and disintegration during the measurements.

To conclude, among all synthesized hydrogels, P(OEGMA_40_/OPGMA_60_) was identified as the most effective drug carrier for diclofenac sodium. This comonomer ratio induces a volume phase transition temperature near the physiological temperature and reflects mild hypothermic conditions. Also, it demonstrates satisfactory in vitro biocompatibility, sustained drug release, and promising potential for drug uptake and drug release under physiological conditions.

## 4. Materials and Methods

### 4.1. Materials

To create hydrogels, oligo(ethylene glycol) methacrylate (OEGMA) (Sigma-Aldrich, Taufkirchen, Germany, M_w_ = 360 g mol^−1^) and oligo(propylene glycol) methacrylate (OPGMA) (Sigma-Aldrich, Taufkirchen, Germany, M_w_ = 375 g mol^−1^) were used as the primary ingredients. As a crosslinking agent, ethylene glycol dimethacrylate (EGDMA) (Sigma-Aldrich, Taufkirchen, Germany, M_w_ = 198.2 g mol^−1^) was employed. For the synthesis of the hydrogels and the preparation of buffer solutions, demineralized water (demi-water, Millipore, Bedford, MA, USA) and absolute ethyl alcohol were used. Hydrochloric acid (La Chema, Brno, Czech Republic), potassium chloride, potassium mono- and di-hydrogen phosphate, sodium hydroxide, ammonium hydroxide, and ammonium chloride (Fluka, Buchs, Switzerland) were used to prepare buffer solutions with pH values of 2.2, 3.85, 4.5, 5.45, 6.2, 6.8, 7.4, 8.2, and 9.0, all at a constant ionic strength of 0.1 mol dm^−3^. For drug delivery, diclofenac sodium (DS), an active diclofenac ingredient (Galenika, Belgrade, Serbia), was utilized. All the chemicals were used directly as received without further purification.

### 4.2. Polymer Library Synthesis

Monomers were dissolved in water/ethanol (50/50, *v*/*v*) solution by stirring at room temperature for 15 min. The total concentration of monomers in the reaction mixture was 10%. To facilitate the crosslinking of copolymeric hydrogels with higher OEGMA and OPGMA content, an EGDMA was added in minimal amounts to all monomer–solvent mixtures. The reaction mixtures were degassed under nitrogen for a short period and sealed between two equally spaced glass plates. Radiation-induced synthesis was performed in a ^60^Co gamma source under ambient conditions at a dose rate of 0.5 kGy/h up to the total absorbed dose of 25 kGy. After annealing in an oven at 60 °C for 24 h, the specimens were cut into discs and dried to a constant weight. Finally, five different P(OEGMA/OPGMA) copolymeric hydrogels were synthesized with varying monomer feed ratios of OEGMA to OPGMA (75:25, 50:50, 40:60, 30:70, and 15:85), along with two homopolymeric hydrogels, POEGMA and POPGMA, all of which were then ready for further characterization (Table 2).

### 4.3. Gel Content and Sol-Gel Conversion

The gel content is considered to be a quantitative indicator of the efficiency of sol-gel conversion and network formation. The xerogels obtained after synthesis and drying were subjected to Soxhlet extraction at 40 °C in water/ethanol solution for 48 h to remove unreacted components. Afterward, the extracted samples were dried for an additional 72 h at the same temperature in a vacuum oven to achieve constant weight. The following equation was used in gravimetric analysis to determine the gel fraction:

(1) $$Gel\;fraction\left(\%\right)=\frac{W_{e}}{W_{0}}\cdot 100$$

where *W_e_* is the weight of the xerogel after extraction, and *W*_0_ is the initial weight of the xerogel.

For the radiation dose of 25 kGy, which is generally recommended for terminal sterilization of medical products [42], the gel content ranged from approximately 80% for POEGMA to 91% for POPGMA, with the gel content of the copolymeric hydrogels falling between these values. This indicates a successful process of radiation crosslinking and synthesis.

### 4.4. Swelling Study

Swelling measurements were performed over a broad temperature range from 5 to 80 °C in a pH 7.4 buffer solution important for biomedical studies. Since the presence of dissolved ions significantly impacts how alkylene glycol polymers swell, all buffers’ ionic strengths were adjusted to 0.1 mol dm^−3^ by adding KCl [43]. The pH will decrease as the temperature rises because the increase in molecular vibrations makes it easier for water to ionize and produce more hydrogen ions. Buffers were therefore set to the initial pH when pH decreased with temperature by more than 0.2 [44,45].

Gravimetric measurements were conducted to calculate the equilibrium swelling degree (*Q_e_*) and determine the temperature of the hydrogel’s volume phase transition (*VPTT*). The equilibrium swelling degree (*Q_e_*) was calculated as

(2) $$Q_{e}=\frac{W_{e}-W_{0}}{W_{0}}$$

where *W_e_* is the weight of the swollen hydrogel at equilibrium at a given temperature, and *W*_0_ is the weight of the xerogel. The average value was calculated using three different samples of the same hydrogel. To obtain more reliable temperature data, before weight measuring, the xerogel samples were swollen to equilibrium at 5 °C, at which point the temperature was raised in 2.5 °C increments every 24 h, which is the time required to achieve swelling equilibrium for methacrylate hydrogels [46,47,48,49]. Lastly, the *VPTT* was graphically determined by examining the influence of temperature on swelling degree under simulated physiological pH and over a broad temperature range.

Another purpose of gravimetric measurements was to examine the pH sensitivity of synthesized hydrogels. Additionally, these measurements were used to track changes in the *VPTT* with varying pH values of the buffers in which the swelling of the tested hydrogels was performed.

### 4.5. Scanning Electron Microscopy (SEM)

The microstructure of the freeze-dried P(OEGMA_40_/OPGMA_60_) hydrogels was observed under a scanning electron microscope (JEOL JSM-6460 LV, Tokyo, Japan).

The samples were prepared using an Edwards Freeze Dryer System (Crawley, UK) consisting of a freeze-drying unit and an Edwards E2M8 high vacuum pump (Crawley, UK). Firstly, P(OEGMA_40_/OPGMA_60_) xerogel samples were swollen to equilibrium in pH 7.4 buffer solution at 20 and 40 °C. Afterward, the samples were pre-frozen in a freeze dryer at −80 °C, freeze-dried for 24 h in a vacuum, and fixed to a graphite stub and sputter-coated with a 10 nm gold layer in the Polaron E5200 SEM (Polaron Equipment Ltd., Watford, UK) auto-coating sputter system under a low vacuum before SEM measurements [50,51].

### 4.6. Biocompatibility Tests

In this study, the effect of various copolymeric hydrogel compositions on biocompatibility was investigated using in vitro cytotoxicity and hemolytic activity.

#### 4.6.1. In Vitro Cytotoxicity

The cytocompatibility of the copolymeric hydrogels was evaluated using the in vitro cell viability method according to ISO 10993-5 [52,53]. The test was performed on mouse sub-connective tissue cells from the NCTC Clone 929 line (ATCC CCL1) using a diluted extract of the hydrogels. Two-hour incubation was carried out in a water bath at 37 °C. A high-density polyethylene extract and a 0.02 wt% phenol solution were used as negative and positive controls, respectively. The cytotoxic potential of the material is defined by the cytotoxicity index *IC*_50%_, which represents the concentration of the extract that inhibits cell colony formation by 50% when compared to the control [54]. The samples can be classified as non-cytotoxic if the cell viability is higher than 50% [35]. To express the results, the average percentage of viable cells from at least three replicates was calculated.

#### 4.6.2. Investigation of Hemolysis Rate

Hemocompatibility is a crucial factor that limits the clinical applicability of blood-contacting materials [55]. To investigate the hemolysis effect of the hydrogels, direct and indirect contact methods according to ISO 10993-4 [56] were applied.

The direct contact method involved immersing the hydrogel samples in a mixture containing 0.25 mL of whole blood collected from a rat and 5 mL of physiological solution. Physiological solution and distilled water served as negative and positive controls, respectively. The samples were mixed and incubated at 37 °C for 1 h and vortexed afterward.

The indirect contact method combined 5 mL of isotonic aqueous solution extracted from hydrogel discs with 0.25 mL of 10% rat erythrocyte suspension. To make the isotonic aqueous extracts, hydrogel discs were immersed in 100 mL of sterilized demineralized water containing 0.9 g NaCl for 72 h at 37 °C. Physiological solution and distilled water served as negative and positive controls, respectively. After 24 h of incubation at 37 °C, the samples were centrifuged.

The absorbances of sample supernatants obtained by both direct and indirect methods were measured at 545 nm, and the percentage of hemolysis was calculated using Equation (3):

(3) $$Hemolysis\;rate\left(\%\right)=\frac{A_{s}-A_{n}}{A_{p}-A_{n}}\cdot 100$$

where *A_s_*, *A_p_*, and *A_n_* are the absorbances of supernatants of the samples, the positive control sample, and the negative control sample, respectively. The measurements were carried out in triplicate, and the mean value was calculated for each sample.

### 4.7. In Vitro Controlled Release of Diclofenac Sodium from Hydrogel Matrices

The absorption of fluid by a xerogel initiates swelling until equilibrium is reached. This swelling process, propelled by diffusion and capillary penetration of the fluid into the gel network, represents a crucial characteristic of hydrogels, especially in medical applications. Therefore, precise control over fluid diffusion, including solutions carrying active substances, remains of paramount importance.

In the context of hydrogels, diffusion manifests as a phenomenon intricately linked with both fluid concentration and the degree of hydrogel swelling [54]. The transport of fluids or small molecules into or out of the polymer network is governed by diffusion, facilitated by concentration gradients and the relaxation process of polymer chains. Polymer relaxation, being time-dependent, significantly influences the rate and extent of molecular penetration into the polymer network.

To determine the nature of fluid diffusion into and out of the hydrogel network, a semi-empirical equation is used [57]:

(4) $$\frac{M_{t}}{M_{\infty }}=k\cdot t^{n}$$

where *k* is the swelling kinetic constant dependent on the hydrogel’s geometry, and *n* is the diffusion exponent, indicating whether the swelling process is controlled by diffusion and/or network relaxation. *M_t_* and *M_∞_* represent the masses of the hydrogel at time *t* and equilibrium, respectively.

In the linear dependence of ln(*M_t_*/*M_∞_*) on ln*t* (Equation (5)), which corresponds to the initial phase of hydrogel swelling as determined by logarithmically transforming Equation (4), the value of ln*k* represents the intercept on the ordinate, while *n* is the slope of the line [58].

(5) $$\ln\frac{M_{t}}{M_{\infty }}=lnk+n\cdot lnt$$

The diffusion exponent (*n*) defines the type of fluid transport in the hydrogel. For *n* ≤ 0.5 in the case of one-dimensional plate geometry, diffusion is known as Fickian diffusion or quasi-Fickian diffusion, which is characterized by the fact that the drug molecules are predominantly governed by the molecular motion within the polymer structure and that the drug release rate is time-dependent. This can happen when the drug molecules are small enough to move relatively freely through the polymer matrix or when the polymer network is relatively porous, allowing for easy diffusion.

For *n* = 1, the transport mechanism significantly diverges from Fickian behavior, with the relaxation rate of the polymer network either comparable to or exceeding the diffusion rate of the drug molecules. This results in a complex release behavior where the drug molecules not only diffuse through the polymer matrix but also experience significant interactions with the polymer chains. Such a mechanism of diffusion is called non-Fickian diffusion. For values 0.5 < *n* < 1, both the diffusion through the polymer network and the relaxation of the polymer chains contribute significantly to the overall transport process. Such transport deviates from Fick’s law and is called anomalous diffusion [58].

The kinetics governing the release of active substances from hydrogels are influenced by several factors, including their chemical composition, structural properties, fabrication methods, geometry, external environmental conditions during release, and the molecular characteristics of the active compound. Various release profiles exist, particularly in hydrogel matrices. One such profile delineates two distinct phases: an initial phase known as external diffusion, characterized by rapid release or the “burst effect”, followed by a subsequent phase termed internal diffusion, which entails slower release. During the early phase, the active substance is primarily liberated from the hydrogel’s surface and near-surface layers, constituting approximately 60% of the release process. Conversely, the late phase, encompassing the remaining 40%, involves diffusion within the hydrogel pores [59]. For the early stage of the release process, the following equation is used:

(6) $$\frac{M_{t}}{M_{\infty }}=4\cdot {\left(\frac{D_{e}\cdot t}{\pi \cdot l^{2}}\right)}^{\frac{1}{2}}\;0<\frac{M_{t}}{M_{\infty }}<0.6$$

This equation illustrates the ratio of the drug amount released in time *t* (*M_t_*) to the total released amount (*M_∞_*) over a shorter time interval [60,61]. *D_e_* denotes the diffusion coefficient of the fluid during the early phase, while *l* represents the sample thickness. By analyzing the slope of the curve *M_t_/M_∞_* plotted against *t*^1/2^, one can determine the diffusion coefficient during the initial phase of active substance release for a specific hydrogel [62].

To describe the late phase of drug release, the following relation [61] is employed:

(7) $$\frac{M_{t}}{M_{\infty }}=1-\frac{8}{\pi^{2}}\cdot exp\left(\frac{-\pi^{2}\cdot D_{l}\cdot t}{l^{2}}\right)\;\;\;\;\;\;\;\;\;\;\;0.6<\frac{M_{t}}{M_{\infty }}<1$$

This equation allows for the calculation of the diffusion coefficient for the late phase, *D_l_*, determined by the slope of the curve ln(1 − *M_t_*/*M_∞_*) plotted against *t* [58,60,62]. This method yields accurate results primarily in systems where diffusion predominates as the drug-release mechanism.

The DS-loaded hydrogels were prepared using the permeation technique, which consists of placing a fully formed xerogel into a medium saturated with the drug, prepared by dissolving drug powder in demineralized water [63]. Xerogels of similar shapes and masses, with various contents of OPGMA and OEGMA monomers, were immersed in a 2 mg mL^−1^ DS solution (concentration of initial loading solution) at 5 °C for two days. The DS-absorbed hydrogels were removed from the solution after reaching an equilibrium swelling of the hydrogel matrices and allowed to dry to a constant mass at room temperature in the absence of light. Following this, the drug-loaded xerogels were employed in a controlled drug delivery experiment.

The analysis of the controlled release of the DS from the xerogel matrices was performed under in vitro conditions. Xerogels containing the absorbed drug were placed in 30 mL of pH 7.4 buffer solution on a magnetic stirrer with heating under constant stirring (200 rpm) and a digital thermostat set at 37 °C, simulating the human physiological environment. The amount of released drug was monitored on a Shimadzu 1800 UV-vis spectrophotometer (Shimadzu Corp., Kyoto, Japan) for 7 days by measuring the absorbance of aliquots of the solution at predetermined time intervals. After each measurement, an aliquot was returned to the buffer solution to maintain the volume of the solution constant. To monitor the release of the drug, absorbances were measured at 276 nm, a wavelength corresponding to one of the DS chromophores (2,6-dichlorodiphenylamine) susceptible to UV-Vis spectrophotometric measurements [64,65]. Before analysis, calibration was made with a series of DS standard solutions in pH 7.4 buffer solution at 276 nm. A calibration graph for DS was plotted in the range of 5–200 μM. A linear equation *A* = 10,300·*c* + 0.01 (where *A* is absorbance of the drug, and *c* is the molar drug concentration) was obtained from the calibration curve with a correlation coefficient R^2^ > 0.995 (for nine points). All measurements were made in triplicate, and all triplicate measurements did not show significant deviations (slope, *a* = (1.03 ± 0.02) × 10^4^ dm^3^ mol^−1^ and y-intercept, *b* = 0.01 ± 0.01), which confirmed good repeatability. The results of the release experiments were analyzed using OriginPro 9.0 software.

## Figures and Tables

**Table 1:** Equilibrium swelling degree (*Q_e_*) and drug release kinetic parameters for POEGMA, P(OEGMA_75_/OPGMA_25_), P(OEGMA_50_/OPGMA_50_), P(OEGMA_40_/OPGMA_60_), P(OEGMA_30_/OPGMA_70_), P(OEGMA_15_/OPGMA_85_), and POPGMA hydrogel matrices. The kinetic parameters are the kinetic constant (*k*), diffusion exponent (*n*), diffusion coefficients for the early (*D_e_*) and late (*D_l_*) stages of drug release, and drug release half-time (*t*_1/2_).

Hydrogel Composition (mol%) P(OEGMA/OPGMA)	*Q_e_* (37 °C)	*k* (h^−*n*^)	*n*	*D_e_* × 10^−7^ (cm^2^/s)	*D_l_* × 10^−8^ (cm^2^/s)	*D_e_*/*D_l_*	*t*_1/2_ (h)
100/0	7.10	0.38	0.50	3.1	3.4	9.1	1.7
75/25	4.40	0.33	0.49	2.3	2.7	8.5	2.3
50/50	1.30	0.27	0.47	1.4	2.0	7.0	3.9
40/60	0.65	0.20	0.43	0.7	1.7	4.1	8.5
30/70	0.40	0.17	0.41	0.4	1.4	2.9	18.9
15/85	0.25	0.15	0.40	0.3	1.3	2.3	29.1
0/100	0.15	0.13	0.39	0.25	1.2	2.1	38.8

**Table 2:** Hydrogel composition, monomer content, and designations of investigated hydrogels.

Hydrogel Samples	Monomer Content		Hydrogel Designation
	OEGMA (mol%)	OPGMA (mol%)	
POEGMA	100	0	POEGMA
P(OEGMA/OPGMA)	75	25	P(OEGMA_75_/OPGMA_25_)
	50	50	P(OEGMA_50_/OPGMA_50_)
	40	60	P(OEGMA_40_/OPGMA_60_)
	30	70	P(OEGMA_30_/OPGMA_70_)
	15	85	P(OEGMA_15_/OPGMA_85_)
POPGMA	0	100	POPGMA

## Data Availability

The original contributions presented in this study are included in the article. Further inquiries can be directed to the corresponding author.

## Abbreviations

The following abbreviations are used in this manuscript:

OEGMA	Oligo(ethylene glycol) methacrylate.
OPGMA	Oligo(propylene glycol) methacrylate.
POEGMA	Poly oligo(ethylene glycol) methacrylate.
POPGMA	Poly oligo(propylene glycol) methacrylate.
P(OEGMA/OPGMA)	Poly (oligo(ethylene glycol) methacrylate/oligo(propylene glycol) methacrylate).
VPTT	Volume phase transition temperatures.
PEG	Poly(ethylene glycol).
PEGMA	Poly(ethylene glycol) methacrylate.
PPG	Poly(propylene glycol).
PPGMA	Poly(propylene glycol) methacrylate.
EG	Ethylene glycol.
PG	Propylene glycol.
SEM	Scanning electron microscopy.
DS	Diclofenac sodium.
EGDMA	Ethylene glycol dimethacrylate.
